# Clinical Utility of Increased Nuchal Translucency at 11–13 Weeks of Gestation in Twin Pregnancies Based on the Chorionicity

**DOI:** 10.3390/jcm10030433

**Published:** 2021-01-23

**Authors:** SiWon Lee, Hyun-Mi Lee, You Jung Han, Moon Young Kim, Hye Yeon Boo, Jin Hoon Chung

**Affiliations:** 1 Department of Obstetrics and Gynecology, Mount Sinai Medical Center, Miami Beach, FL 33109, USA; c1loveya@gmail.com; 2 Department of Obstetrics and Gynecology, CHA Ilsan Medical Center, CHA University, Goyang 10414, Korea; hmlee984415@gmail.com (H.-M.L.); lrhnb4@gmail.com (H.Y.B.); 3 Department of Obstetrics and Gynecology, CHA Gangnam Medical Center, CHA University, Seoul 06135, Korea; hanyj1978@gmail.com (Y.J.H.); mykimdr@gmail.com (M.Y.K.); 4 Department of Obstetrics and Gynecology, Asan Medical Center, University of Ulsan College of Medicine, Seoul 05505, Korea

**Keywords:** twin pregnancy, nuchal translucency, chorionicity, anomaly, twin complications

## Abstract

To assess clinical implications of increased nuchal translucency (INT) in twin pregnancies based on the chorionicity. This was a retrospective review of the twin pregnancies who underwent first trimester ultrasound with nuchal translucency (NT) measurement at 11–13 weeks of gestation from January 2006 to December 2014. Data were collected using the OB database and the chart review. Pregnancy outcomes, including gestational weeks at the delivery, abnormal fetal karyotypes, fetal structural anomalies, and twin-specific complications, were analyzed. A total of 1622 twin pregnancies with INT ≥ 95th percentile in one or both fetuses were identified. In all twin pregnancies with INT, abnormal fetal karyotypes were identified in 17 (8.6%) patients (odds ratio = 13.28, CI = 5.990–29.447, *p* = 0.000) and twin-specific complications were identified in 23 (11.6%) patients (odds ratio = 2.398, CI = 1.463–3.928, *p* = 0.001) compared to those with normal NT. Among the INT group, when the groups were subdivided into monochorionic (MC) and dichorionic (DC) pregnancies, 14.8% and 29.6% of the MC pregnancies had structural anomalies in one or both fetuses (odds ratio = 5.774, 95% CI = 1.445–23.071, *p* = 0.01) and twin-specific complications (odds ratio = 4.379, 95% CI = 1.641–11.684, *p* = 0.03), respectively, compared to DC pregnancies with 2.9% for structural anomalies and 8.8% for twin-specific complications. The prevalence of abnormal fetal karyotypes was not statistically different in patients with INT when compared between MC and DC pregnancies (*p* = 0.329). INT was associated with a higher rate of twin-specific complications and fetal structural anomalies in MC twin pregnancies rather than abnormal fetal karyotype. Therefore, NT measurement in MC twin pregnancies can be a useful tool for predicting adverse pregnancy outcomes. Appropriate counseling and surveillance based on the chorionicity are imperative in the prenatal care of twin pregnancies.

## 1. Introduction

The incidence of multiple gestations is rising, due to an increased rate of assisted reproductive techniques (ART) and advanced maternal age [1]. The risk of chromosomal abnormalities is also increasing because of advanced maternal age as a result of delayed childbearing [2].

Options for the aneuploidy screening in twin pregnancies are similar to those performed in singleton pregnancies, including nuchal translucency measurement, maternal serum screening test, and cell-free DNA (cfDNA) test. However, interpretation of the maternal serum screening test is challenging in multiple pregnancies, and the detection rate (DR) of triple and quadruple screening test was reported to be about 44% and 47%, respectively, in twin pregnancies [3]. In recent years, cfDNA was introduced for aneuploidy screening in twin pregnancies, and some studies reported that the performance of cfDNA testing for trisomy 21 in twin pregnancies is similar to that in a singleton pregnancy and is superior to that of the combined maternal serum screening tests [4,5]. However, the American College of Obstetricians and Gynecologists does not currently recommend offering cfDNA testing to women with multiple gestations because of the lack of validation [4,6,7]. In other words, none of the serum aneuploidy screening tests currently available are proven to be as accurate in twin pregnancies as it is with singleton pregnancies.

In singleton pregnancy, nuchal translucency measurement in the first trimester is included as part of the routine prenatal care in many places for aneuploidy screening, as well as structural anomalies and adverse pregnancy outcomes in fetuses with normal karyotypes [8,9,10]. In twin pregnancies, DR of trisomy 21 based on increased nuchal translucency (INT) was reported to be as high as 90%, with a 5% positive predictive value [11,12]. In monochorionic (MC) twin pregnancies, INT is also correlated with the twin-specific complications, such as twin-twin transfusion syndrome (TTTS) [13]. However, only a few studies have provided the information available regarding the value of INT based on the chorionicity and related unique complications in twin pregnancies [8].

The objective of this study was to analyze the significance of INT in one or both fetuses at the first trimester scan in twin pregnancies based on the chorionicity to predict adverse pregnancy outcomes.

## 2. Materials and Methods

This was a retrospective study conducted at Cheil General Hospital and Women’s Healthcare Center, Seoul, South Korea, from January 2006 to December 2014. All twin pregnancies who underwent first trimester ultrasound with NT screening at 11–13 weeks of gestation with complete prenatal care and known pregnancy outcomes were included. Data were collected using the OB database and the chart review. Pregnancy outcomes, including gestational weeks at the delivery, abnormal fetal karyotypes, fetal structural anomalies, and twin-specific complications, were analyzed. Ethical approval of the study was obtained from the institutional review board (CGH-IRB-2014-5). The need to obtain informed consent was waived.

NT measurement was carried out by three maternal-fetal medicine specialists or five registered diagnostic medical sonographers accredited by either Fetal Medicine Foundation or Nuchal Translucency Review Program. NT was measured using transabdominal or transvaginal ultrasonography in a good mid-sagittal section of the fetus with magnification such that the fetus occupied at least 75% of the image. NT was measured at least three times during the scan, and the largest of the three measurements was selected for analysis. Gestational age was determined from the first day of the last menstrual period (LMP) and was confirmed by crown rump length (CRL) of the larger twin in the first trimester of pregnancy [14]. When there were more than five days of discrepancy between LMP and CRL, we used the gestational age based on CRL at 7–9 weeks of gestation. In cases that pregnancy was achieved by in vitro fertilization and embryo transfer (IVF-ET), ovum retrieval date was used as 2 + 0 weeks of gestation. We defined INT as a measurement greater than the 95^th^ percentile for each fetal CRL using the reference value previously published from our institution [15]. Chorionicity was determined by assessing the number of the gestational sac, yolk sac, and fetus at 7–9 weeks of gestation and the shape of the intertwin membranes in the ultrasound examination. Chorionicity was confirmed with the histologic examination of the postpartum placenta [16].

Pregnancy outcomes were obtained from the medical records of the mother and the infant. As cfDNA was not introduced in Korea at the time of this study period, fetal karyotyping was offered to the patients by following indications in addition to routine indications: Maternal age ≥31 years in dichorionic (DC) twins, maternal age ≥35 years in monochorionic (MC) twins, and detection of fetal structural anomaly or INT in the first trimester ultrasound. Fetal karyotyping was performed with either amniocentesis or chorionic villus sampling. Neonates or stillborn babies without karyotyping results were examined after delivery for phenotypic abnormalities indicating chromosomal aberrations.

Twin-specific complications included twin-twin transfusion syndrome (TTTS), discordant twins, selective intrauterine growth restriction (sIUGR), co-twin or both twin demises, acardiac twins, and conjoined twins. Patients with acardiac twins and conjoined twins were excluded from this study because accurate NT measurements were impossible in these cases. Diagnosis of TTTS was made according to the established criteria by Quintero et al. [17]. Criteria included MC twin and discordant amniotic fluid volume (AFV), with MVP of more than 8 cm in the recipient twin and MVP of less than 2 cm in the donor twin. The sIUGR was defined when estimated fetal weight (EFW) fell below the 10^th^ percentile in one of the MC twins, while the co-twin was of normal size and discordant AFV did not meet the criteria seen in TTTS [18]. We defined discordant twins as greater than 25% difference in EFW between the fetuses in DC twin [19]. Structural anomalies were described only in fetuses with normal karyotype.

Statistical analysis was done with the Statistical Package for Social Sciences version 16.0 (SPSS Inc., Chicago, IL, USA). Continuous variables were presented as mean ± standard deviation (SD) and were compared using Student’s t-test. Categorical data were presented as *n* (%) and analyzed using the chi-square test and Fisher’s exact test. In all the tests, a *p*-value < 0.05 was considered statistically significant.

## 3. Results

**Figure 1:**
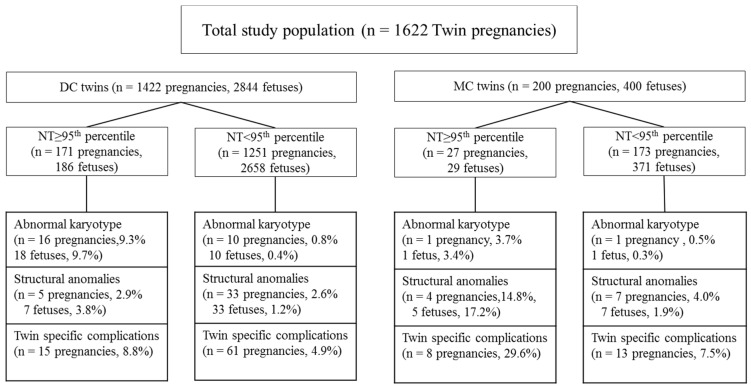
Flow chart summarizing the clinical outcomes of the study population (*n* = 1622). DC, dichorionic; MC, monochorionic; NT, nuchal translucency.

We identified 1839 twin pregnancies that underwent first trimester ultrasound at 11–13 weeks of gestation. We excluded four pregnancies without NT measurements, and 213 pregnancies that were lost to follow-up. A total of 1622 twin pregnancies were finally included in the analysis (Figure 1).

Table 1 shows the demographic characteristics and outcomes of all study populations. Of the 1622 patients with twin pregnancies included in this study, 1422 (87.7%) patients were DC pregnancies, and 200 (12.3%) patients were MC pregnancies. INT in one or both fetuses were detected in 198 (12.2%) out of 1622 pregnancies, and a total of 215 (6.6%) individual fetuses out of 3244 fetuses had INT. Abnormal fetal karyotype was identified in 27 pregnancies (1.7%, 27/1622), and 3 of them (2 DC pregnancies and 1 MC pregnancy) exhibited abnormal karyotype in both fetuses, yielding a total of 30 fetuses with chromosomal abnormalities (0.9%, 30/3244). Out of the 215 fetuses with INT, 19 fetuses had abnormal karyotype (8.8%, 19/215). In one MC twin pregnancy with trisomy 13, fetus A had a normal NT of 2.1 mm, whereas fetus B had INT of 9.1 mm. Two patients with DC twins had abnormal karyotypes in both fetuses. The first patient with DCDA pregnancy who had INT of 11.5 mm and 2.6 mm in each fetus, revealed 45,XO and 46,inv(2)(p11.2;q13), respectively. The second patient with DCDA pregnancy who had INT of 7.9 mm and 7.3 mm in each fetus, revealed trisomy 13 and trisomy 18, respectively. The remaining cases with abnormal karyotypes included 6 fetuses with trisomy 21, 1 fetus with trisomy 18, 3 fetuses with 45,XO, and 1 each of 47,XYY, 47,XXY, 47,XX marker chromosome and 48XXY,+12/46,XY.

The fetal structural anomaly was noted in 49 twin pregnancies (3.0%, 49/1622). Among those patients, 3 had single or multiple structural anomalies in both fetuses, yielding a total of 52 fetuses (1.6%, 52/3244) with structural anomalies. The structural anomalies included 19 fetuses with central nervous system (CNS) anomalies (ex. Ventriculomegaly, Dandy-Walker malformation, acrania), 12 fetuses with urinary tract anomalies (ex. Ureteropelvic junction (UPJ) obstruction, horseshoe kidney, and renal agenesis), 11 fetuses with congenital heart anomalies (ex. Tetralogy of Fallot (TOF), ventricular septal defect (VSD) and coarctation of the aorta (COA)) and 10 fetuses with other anomalies (ex. Hydrops fetalis, cleft lip and palate, adrenal mass, Limb-body-wall complex, hemivertebra, porto-hepatic shunt and club foot).

Twin-specific complications were identified in 97 twin pregnancies (6.0%, 97/1622). Discordant twins were identified in 76 (5.3%, 76/1422) of DC twin pregnancies. Out of 200 MC twin pregnancies, TTTS was identified in sIUGR in 8 (4.0%) patients, 7 (3.5%) patients, and co-twin or both twin demises were identified in 6 (3.0%) patients.

Comparison of all twin pregnancies with INT and normal NT are shown in Table 2. There were no significant differences in maternal age, parity, conceptional method, gestational age at NT measurement, and chorionicity between the two groups. However, in the INT group, 17 out of 198 patients (8.6%) had abnormal fetal karyotypes (odds ratio = 13.28, CI = 5.990–29.447, *p <* 0.0001) and 23 out of 198 patients (11.6%) had twin-specific complications (odds ratio = 2.398, CI = 1.463–3.928, *p* = 0.001) which were statistically significant compared to those with normal NT. The prevalence of fetal structural anomalies was not significantly different between the two groups (*p* = 0.183). When individual fetuses with INT and normal NT were compared, abnormal karyotype and structural anomalies were significantly higher in INT group (both *p* < 0.00001).

Comparison of demographic characteristics and pregnancy outcomes in DC and MC pregnancies are shown in Table 3. Maternal age was significantly higher in DC pregnancies compared to that of MC pregnancies (*p* = 0.000), as was the frequency of assisted reproductive technology (ART) and nulliparity (*p* = 0.000). The prevalence of INT and abnormal fetal karyotypes were not statistically different between the two groups. The prevalence of fetal structural anomalies (odds ratio = 2.120, 95% CI = 1.065–4.218, *p* = 0.043) and twin-specific complications were significantly higher in MC twins (odds ratio = 2.078, 95% CI = 1.251–3.452, *p* = 0.007). Among the 1422 DC twin pregnancies, 12.0% had INT, and 13.5% of the 200 MC twin pregnancies had INT (*p* = 0.240). When the pregnancy outcomes were analyzed and compared in the individual fetuses between the groups, no differences in the prevalence of INT, abnormal karyotypes, or structural anomalies were noted.

Table 4 compares pregnancy outcomes between INT ≥ 95^th^ percentile and normal NT in DC twin pregnancy (*n* = 1422). Incidence of abnormal fetal karyotype (9.3% vs. 0.8%, *p* < 0.001, odds ratio = 12.81, 95% CI = 5.71–28.72) and twin-specific complications (8.8% vs. 4.9%, *p* = 0.0036, odds ratio = 1.88, 95% CI = 1.04–3.38) were significantly higher in pregnancies with INT ≥ 95^th^ percentile compared to normal NT. No statistical difference was noted in pregnancies with fetal structural anomalies between the two groups (8.8% vs. 4.9%, *p* = 0.828, odds ratio = 1.11, 95% CI = 0.43–289). However, when individual fetuses were analyzed between INT ≥ 95^th^ percentile vs. normal NT groups, significant differences were noted not only in fetuses with abnormal karyotype (9.7% vs. 0.4%, *p* = 0.00002, odds ratio = 8.48, 95% CI = 2.76–26.11), but also in fetuses with structural anomalies (3.8% vs. 1.2%, *p* = 0.0073, odds ratio = 3.11, 95% CI = 1.36–7.13). In DC twin pregnancies, twin-specific complications included discordant twins. In chromosomally normal fetuses with INT in one or both twins, 76 discordant twins (5.3%, 76/1422) were shown. When subdivided into INT and normal NT, the incidence of discordant twins in each group were 8.8% vs. 4.9%, respectively (*p* = 0.0036).

Table 5 compares pregnancy outcomes between INT ≥ 95^th^ percentile and normal NT in MC twin pregnancy (*n* = 200). There was no difference between the group in terms of abnormal fetal karyotype (3.7% vs. 0.5%, *p* =.1863, odds ratio = 6.62, 95% CI = 0.40–10.9.04). However, significant differences were noted on the incidence of pregnancies with fetal structural anomalies (14.7% vs. 4.0%, *p* = 0.0331, odds ratio = 4.12, 95% CI = 1.12–15.19) and pregnancies with twin-specific complications (29.6% vs. 7.5%, *p* = 0.0013, odds ratio= 5.18, 95% CI = 1.9–14.11) when compared between INT ≥ 95^th^ percentile vs. normal NT groups. Likewise, when individual fetuses were analyzed between INT ≥ 95^th^ percentile vs. normal NT groups, no difference was noted on the incidence of abnormal fetal karyotype (3.4% vs. 0.3%, *p* = 0.0706, odds ratio = 13.2, 95% CI = 0.80–216.94), but significantly higher incidence of fetuses with structural anomalies was noted in INT ≥ 95^th^ percentile group compared to normal NT group (17.2% vs. 1.9%, *p* = 0.0001, odds ratio = 10.83, 95% CI = 3.20–36.68). Of note, we had only one case of abnormal karyotype in MC twin pregnancy group, with one fetus having abnormal NT of 9.1 mm and the other with normal NT. We performed CVS in the first trimester, which resulted in trisomy 13. To confirm the karyotype of each fetus, we consequently performed amniocentesis in each fetus, and both fetuses were confirmed to have trisomy 13.

The total twin-specific complications in our study in MC twin pregnancy included 8 cases of sIUGR (4.0%), 7 cases of TTTS (3.5%), and 6 cases of co-twin or both twin demise (3.0%). Out of these cases, INT ≥ 95^th^ percentile in one or both fetuses was noted in 3 cases of sIUGR, 3 cases of TTTS, and 2 cases of fetal demise.

Table 6 compares pregnancy outcomes between DC and MC only in pregnancies and fetuses with INT. Out of 27 MC pregnancies with INT, 14.8% had structural anomalies (odds ratio = 5.774, 95% CI = 1.445–23.071, *p* = 0.01), and 29.6% had twin-specific complications (odds ratio = 4.379, 95% CI = 1.641–11.684, *p* = 0.03) which were statistically higher compared to DC twin pregnancies with 2.9% for structural anomalies and 8.8% for twin-specific complications. There was only one case of abnormal fetal karyotype in MC pregnancies, however, the prevalence was not different between DC and MC twins (*p* = 0.329). When individual fetuses with INT were compared between DC and MC groups, structural anomalies were significantly higher in MC group (odds ratio = 5.327, 95% CI = 1.566–18.121, *p* = 0.007).

## 4. Discussion

This study demonstrates that NT screening in the first trimester of pregnancy plays an important role in twin pregnancies not only for the aneuploidy screening, but also for predicting the twin-specific complications and structural anomalies. Several researchers have reported a comparison of INT versus normal NT in either DC or MC twins [20,21], however, those studies did not focus on the pregnancy outcomes in relation to INT in twin pregnancies based on the chorionicity. When the pregnancy outcomes in patients with INT were compared between DC and MC twin pregnancies, as in this study, MC twins with INT had a higher incidence of structural anomalies and twin-specific complications than the DC twins with INT implementing different clinical significance of INT in DC and MC twins. Association between INT and twin-specific complications, such as TTTS, were reported in previous studies [13,22], but the relevance was not consistent [23,24]. This study compared twin-specific complications separately in DC and MC twin pregnancies.

In DC twin pregnancies, chromosomally normal fetuses with INT in one or both twins demonstrated a significantly higher incidence of discordant twin in INT group (*p* = 0.0036, odds ratio = 1.88, 95% CI 1.04–3.38). Considering the fact that NT thickness is directly correlated with CRL [15], CRL discordance in early pregnancy may be associated with discordant twins.

In MC twin pregnancies, twin-specific complications were also significantly higher in INT group compared to the normal NT group (=0.0013, odds ratio = 5.18, 95% CI = 1.90–14.11). The total twin-specific complications in MC twin pregnancy included 8 cases of sIUGR (4.0%), 7 cases of TTTS (3.5%), and 6 cases of co-twin or both twin demises (3.0%). Out of these, INT ≥ 95^th^ percentile in one or both fetuses were noted in 3 cases of sIUGR, 3 cases of TTTS, and 2 cases of fetal demise. We have also calculated the delta value of NT measurement in each group, and NT differences were noted to be 0.63 mm in sIUGR group, 1.85 mm in TTTS group, and 1.5 mm in the demise group on average, which is in partial agreement with the findings from the recent publication by Cimpoca B. et al., implying that the finding in one or both fetuses with INT ≥ 95^th^ percentile and more so in ≥99^th^ percentile, is associated with a substantially increased risk of fetal loss or need for endoscopic laser surgery, due to TTTS [8]. Therefore, this study provides data supporting an increased risk of twin-specific complications, especially in MC twin pregnancies with INT, suggesting the importance of early recognition, differentiated counseling, and close surveillance in MC twins.

It is well known that the MC twins have a higher risk of adverse pregnancy outcomes compared to DC twins because of their unique vascular anastomosis that causes hemodynamic imbalances, which are most likely the cause of INT in MC twins [22]. Other possible mechanisms of INT include cardiac dysfunction, venous congestion in the fetal head and neck, alterations in the local extracellular matrix, lymphatic vessel hyperplasia, congenital infection, anemia, and hypoproteinemia [2,9,23]. Twin-to-twin transfusion-associated circulation imbalances are also a possible mechanism of INT in monozygotic monochorionic twins [13]. In previous reports, the prevalence of INT was higher in MC twins compared to DC twins, and it has been shown that NT thickness was 1.5 times greater in MC twins [2]. However, in our study, the prevalence of INT did not differ significantly between DC and MC twins (*p* = 0.564), probably due to an insufficient number of MC twins. Total 9 cases of trisomy 21 were identified in this study, 67% of which were DC twins with INT. This study had only one patient with confirmed trisomy 13 in MC pregnancy, with an INT of 9.1 mm, but interestingly, the other co-twin had normal NT (NT = 2.1 mm). There is some technical difficulty associated with second trimester ultrasound examination in twin pregnancies compared with singletons, but the DR for trisomy 13 by second trimester ultrasound is still above 90%. Thus, there is more concern for fetal structural anomalies and twin-specific complications than chromosomal abnormalities in MC twins when INT is detected in the first trimester.

The main limitation of the present study is that this was retrospective, and 213 cases were inadvertently lost to follow-up. Many of our patients from rural areas who conceived via assisted reproductive technique returned to their areas after the first trimester ultrasound, which explains the large number of lost to follow up. In addition, the numbers of MC twin pregnancies with adverse pregnancy outcomes, including abnormal fetal karyotype, structural anomalies, and twin-specific complications, were relatively small compared to the DC pregnancies. Therefore, future large, randomized, controlled studies will be necessary to further elucidate the clinical implications of INT in MC twin pregnancies. Nevertheless, the main strength of this study is that we were able to include a large number of twin pregnancies that had consistent prenatal care from the first trimester of pregnancy, had complete prenatal care, and delivered in a single institution. This allowed us to collect and analyze using the most accurate data on pregnancy and neonatal outcomes.

## 5. Conclusions

In conclusion, our data suggest that INT in the first trimester ultrasound in twin pregnancy should be considered in different aspects based on the chorionicity. INT in twin pregnancy is associated with not only abnormal fetal karyotypes, but also structural anomalies and twin-specific complications. Our study emphasizes that INT in DC twin pregnancies correlates with the increased incidence of abnormal fetal karyotype, which is similar to that of singleton pregnancies based on individual NT measurement. In addition, an increased incidence of discordant twins was demonstrated in DC pregnancies with discordant NT measurement. In MC twins with INT, significantly more twin-specific complications and structural anomalies were noted, and no significant difference was noted for chromosomal abnormalities. The clinical implication of this study is that detection of INT in twin pregnancies, especially in MC twins with no chromosomal or major structural abnormalities, is important in the early prediction of twin-specific complications. Although appropriate intervention in cases of INT in MC twin pregnancies remains controversial to prevent adverse pregnancy outcomes, determining chorionicity and NT measurement in the first trimester of pregnancy can be a useful tool for early recognition, individualized counseling, and the need for close surveillance.

## Figures and Tables

**Table 1:** Demographic characteristics and pregnancy outcomes in all twin pregnancies (*n* = 1622) and individual fetuses (*n* = 3244).

**Maternal Characteristics**	**Total Pregnancies (*n* = 1622)**
Age, years (mean ± SD)	33.2 ± 3.3
Nulliparity, *n* (%)	1335 (82.3)
Natural pregnancy, *n* (%)	340 (21.0)
GA at NT exam, weeks (mean ± SD)	12.5 ± 0.5
Dichorionic twin, *n* (%)	1422 (87.7)
**Pregnancy outcomes in total twin pregnancies**	***n* = 1622**
Pregnancies with INT, *n* (%)	198 (12.2)
Pregnancies with abnormal fetal karyotypes, *n* (%)	27 (1.7)
Pregnancies with fetal structural anomalies, *n* (%)	49 (3.0)
Pregnancies with twin-specific complications, *n* (%)	97 (6.0)
**Pregnancy outcomes in individual fetuses**	***n* = 3244**
Fetuses with INT, *n* (%)	215 (6.6)
Fetuses with abnormal karyotypes, *n* (%)	30 (0.9)
Fetuses with structural anomalies, *n* (%)	52 (1.6)

GA, gestational age; NT, nuchal translucency; INT, increased nuchal translucency.

**Table 2:** Comparison of Demographic characteristics and pregnancy outcomes in all twin pregnancies and individual fetuses between increased NT ≥ 95^th^ and normal NT.

**Characteristics and Outcomes**	**NT ≥ 95^th^ (*n* = 198)**	**NT < 95^th^ (*n* = 1424)**	***p***	**OR (95% CI)**
Maternal age, years (mean ± SD)	33.4 ± 3.2	33.2 ± 3.3	0.476	
Nulliparity, *n* (%)	163 (82.3)	1172 (82.3)	1.000	
Natural pregnancy, *n* (%)	40 (20.2)	300 (21.1)	0.852	
GA at NT exam, weeks (mean ± SD)	12.5 ± 0.6	12.5 ± 0.5	0.493	
Dichorionic twins, *n* (%)	171 (86.4)	1251 (87.9)	0.564	
Pregnancies with abnormal fetal karyotype, *n* (%)	17 (8.6)	10 (0.7)	<0.0001 *	13.28 (5.99–29.45)
Pregnancies with fetal structural anomalies, *n* (%)	9 (4.5)	40 (2.8)	0.183	1.65 (0.80–3.45)
Pregnancies with twin-specific complications, *n* (%)	23 (11.6)	74 (5.2)	<0.001*	2.39 (1.46–3.94)
**Outcomes of Individual Fetuses with INT**	**NT ≥ 95^th^ (*n* = 215)**	**NT < 95^th^ (*n* = 3029)**	***p***	**OR (95% CI)**
Fetuses with abnormal karyotype, *n* (%)	19 (8.8)	11 (0.36)	<0.00001 *	26.59 (12.48–56.67)
Fetuses with structural anomalies, *n* (%)	12 (5.6)	40 (1.3)	<0.00001 *	4.42 (2.28–8.55)

GA, gestational age; NT, nuchal translucency; * *p*-value < 0.05; OR, odds ratio; CI, confidence interval.

**Table 3:** Comparison of demographic characteristics and pregnancy outcomes in all twin pregnancies and individual fetuses based on the chorionicity.

**Characteristics and Outcomes**	**DC (*n* = 1422)**	**MC (*n* = 200)**	***p***	**OR (95% CI)**
Maternal age, years (mean ± SD)	33.4 ± 3.2	32.4 ± 3.9	<0.0001 *	
Nulliparity, *n* (%)	1198 (84.2)	137 (68.5)	<0.0001 *	
Natural pregnancy, *n* (%)	183 (12.9)	157 (78.5)	<0.0001 *	
GA at NT exam, weeks (mean ± SD)	12.5 ± 0.5	12.5 ± 0.6	0.996	
Pregnancies with fetal INT, *n* (%)	171 (12.0)	27 (13.5)	0.564	
Pregnancies with abnormal fetal karyotypes, *n* (%)	26 (1.8)	1 (0.5)	0.240	0.27 (0.04–1.99)
Pregnancies with fetal structural anomalies, *n* (%)	38 (2.7)	11 (5.5)	0.043 *	2.12 (1.07–4.22)
Pregnancies with twin-specific complications, *n* (%)	76 (5.3)	21 (10.5)	0.007 *	2.08 (1.25–3.45)
**Pregnancy Outcomes in Individual Fetuses**	**DC (*n* = 2844)**	**MC (*n* = 400)**	***p***	**OR (95% CI)**
Fetuses with INT, *n* (%)	186 (6.5)	29 (7.3)	0.593	1.12 (0.74–1.68)
Fetuses with abnormal karyotypes, *n* (%)	28 (1.0)	1 (0.3)	0.144	0.25 (0.03–1.86)
Fetuses with structural anomalies, *n* (%)	40 (1.4)	12 (3.0)	0.133	2.17 (1.13–4.17)

DC, dichorionic; MC, monochorionic; GA, gestational age; NT, nuchal translucency; INT, increased nuchal translucency; * *p*-value < 0.05; OR, odds ratio; CI, confidence interval.

**Table 4:** Comparison of pregnancy outcomes in dc twin pregnancies and individual fetuses between increased NT ≥ 95^th^ and normal NT. (*n* = 1422).

**Characteristics and Outcomes**	**NT ≥ 95^th^ (*n* = 171)**	**NT < 95^th^ (*n* = 1251)**	***p***	**OR (95% CI)**
Pregnancies with abnormal fetal karyotype, *n* (%)	16 (9.3)	10 (0.8)	<0.0001 *	12.81 (5.71–28.72)
Pregnancies with fetal structural anomalies, *n* (%)	5 (2.9)	33 (2.6)	0.828	1.11 (0.43–2.89)
Pregnancies with twin-specific complications, *n* (%)	15 (8.8)	61 (4.9)	0.0036 *	1.88 (1.04–3.38)
**Outcomes of individual fetuses with INT (*n* = 2844)**	**NT ≥ 95^th^ (*n* = 186)**	**NT < 95^th^ (*n* = 2658)**	***p***	**OR (95% CI)**
Fetuses with abnormal karyotype, *n* (%)	18 (9.7)	10 (0.4)	0.00002 *	8.48 (2.76–26.11)
Fetuses with structural anomalies, *n* (%)	7 (3.8)	33 (1.2)	0.0073 *	3.11 (1.36–7.13)

DC, dichorionic; NT, nuchal translucency; * *p*-value < 0.05; OR, odds ratio; CI, confidence interval.

**Table 5:** Comparison of pregnancy outcomes in mc twin pregnancies and individual fetuses between increased NT ≥ 95^th^ and normal NT. (*n* = 200).

**Characteristics and Outcomes**	**NT ≥ 95^th^ (*n* = 27)**	**NT < 95^th^ (*n* = 173)**	***p***	**OR (95% CI)**
Pregnancies with abnormal fetal karyotype, *n* (%)	1 (3.7)	1 (0.5)	0.1863	6.62 (0.40–109.04)
Pregnancies with fetal structural anomalies, *n* (%)	4 (14.8)	7 (4.0)	0.0331 *	4.12 (1.12–15.19)
Pregnancies with twin-specific complications, *n* (%)	8 (29.6)	13 (7.5)	0.0013 *	5.18 (1.90–14.11)
**Outcomes of individual fetuses with INT**	**NT ≥ 95^th^ (*n* = 29)**	**NT < 95^th^ (*n* = 371)**	***p***	**OR (95% CI)**
Fetuses with abnormal karyotype, *n* (%)	1 (3.4)	1 (0.3)	0.0706	13.21 (0.80–216.94)
Fetuses with structural anomalies, *n* (%)	5 (17.2)	7 (1.9)	0.0001 *	10.83 (3.20–36.68)

MC, monochorionic; NT, nuchal translucency; * *p*-value < 0.05; OR, odds ratio; CI, confidence interval.

**Table 6:** Comparison of pregnancy outcomes between DC and MC in pregnancies and individual fetuses with INT ≥ 95^th^.

**Outcomes in Pregnancies with INT**	**DC (*n* = 171)**	**MC (*n* = 27)**	***p***	**OR (95% CI)**
Pregnancies with abnormal fetal karyotype, *n* (%)	16 (9.4)	1 (3.7)	0.329	0.38 (0.05–2.93)
Pregnancies with fetal structural anomalies, *n* (%)	5 (2.9)	4 (14.8)	0.001 *	5.77 (1.45–23.07)
Pregnancies with twin-specific complications, *n* (%)	15 (8.8)	8 (29.6)	0.003 *	4.38 (1.64–11.68)
**Outcomes in individual fetuses with INT**	**DC (*n* = 186)**	**MC (*n* = 29)**	***p***	**OR (95% CI)**
Fetuses with abnormal karyotypes, *n* (%)	18 (9.7)	1 (3.4)	0.294	0.33 (0.04–2.59)
Fetuses with structural anomalies, *n* (%)	7 (3.8)	5 (17.2)	0.007 *	5.33 (1.57–18.12)

DC, dichorionic; MC, monochorionic; INT, increased nuchal translucency; * *p*-value < 0.05; OR, odds ratio; CI, confidence interval.

## Data Availability

**Data available on request due to restrictions**: The data presented in this study are available on request from the corresponding author. The data are not publicly available due to privacy.
